# Giant Prolactinoma Resistant to High-Dose Cabergoline in a Young Male Lost to Follow-Up

**DOI:** 10.7759/cureus.95564

**Published:** 2025-10-28

**Authors:** Anoushka Das, Mohammad Adjmal Rummun, Anindya Shams, Ahmed Salman

**Affiliations:** 1 General Practice, Blackpool Teaching Hospitals, Blackpool, GBR; 2 Internal Medicine, Blackpool Teaching Hospitals, Blackpool, GBR; 3 Endocrinology and Diabetes, Blackpool Teaching Hospitals, Blackpool, GBR; 4 Endocrinology, Diabetes and Metabolism, Blackpool Teaching Hospitals, Blackpool, GBR

**Keywords:** cabergoline, giant prolactinoma, impulse control disorder, macroadenoma, pituitary adenoma

## Abstract

Pituitary adenomas are neoplasms of the adenohypophysis (anterior pituitary gland) that can cause a multitude of symptoms secondary to the hormones they secrete (functioning adenomas) or mass effect on the optic chiasma (non-functioning adenomas). Functioning adenomas can secrete prolactin, growth hormone, adrenocorticotrophic hormone (ACTH) or thyroid-stimulating hormone, with some adenomas co-secreting more than one. This can lead to symptoms of prolactinoma, acromegaly, Cushing’s disease, and TSHoma dependent on the elevated hormone. In non-functioning adenomas, visual changes, and headaches are seen. This case report follows a 19-year-old male who presented with visual acuity loss and persistent headaches, later diagnosed as a pituitary macroadenoma with a prolactin level of 794,560 mIU/L. He was subsequently treated with off-label cabergoline over three times the therapeutic limit for approximately 10 years. He was unfortunately lost to follow-up and unmonitored during this time, where he developed an impulse control disorder and showed inadequate response to the high-dose cabergoline. At the time of re-presenting to outpatient clinics, his adenoma was found to be extending into the cavernous sinuses with tentorial involvement, reducing the potential for surgical management. Discussions around medical, surgical, and radiotherapeutic management are being raised in light of this cabergoline resistance and placement. The case overall highlights the complexities of managing giant, dopamine agonist-resistant prolactinomas, especially in the setting of delayed follow-up, incomplete biochemical control, and neuropsychiatric side effects.

## Introduction

The presentation of pituitary adenoma is highly varied and dependent on a few of its defining features. Approximately 50% are microadenomas, defined as <10 mm; anything larger is considered a macroadenoma with >40 mm being further classified as ‘giant’ ​[1,2]​. They are also categorised by their production of hormones (functioning) or lack thereof (non-functioning), with 40% of pituitary adenomas secreting prolactin ​[3]​. The presentation of functioning adenomas is widely varied in accordance with the rise of the elevated pituitary hormone ​[2]​. As a byproduct of mass effect, both functioning and non-functioning adenomas can result in visual changes, the most common presentation in macroadenomas. A study in 2016 found 40%-60% of patients with pituitary macroadenomas experienced visual changes; the classical bitemporal pattern being the most common ​[4]​ and a retrospective cohort study in 2023 confirmed tumours with >16 mm suprasellar extension were associated with moderate to severe vision loss ​[5]​. Whilst adenomas can commonly extend into the suprasellar cistern, on rarer occasions, they can extend into the cavernous sinuses, sphenoid sinus, and temporal and frontal lobes ​[6]​.

The first-line treatment of a giant prolactinoma is widely accepted to be medical, with cabergoline being the dopamine agonist (DA) of choice ​[7]​. Resistance to DA therapy (failure to achieve normoprolactinemia) is unfortunately well documented and seen in 10% of patients receiving cabergoline [8]. DA therapy also has a well-established risk of developing impulse control disorders (ICDs) ​[9,10]​, with a prevalence study in 2020 further identifying significantly higher incidences of ICDs in patients on DA therapy when compared to a control (61.1% vs 42.4%) ​[11]​.

This case describes the unlicenced use of high-dose cabergoline (14 mg/week) to treat a macroprolactinoma in a patient lost to follow-up through disengagement with clinics. The consequences of this led to the development of disordered impulse control severely impacting on the patient’s quality of life and the delayed recognition of resistance to cabergoline treatment. Pituitary macroadenomas are a nuanced condition with multiple treatment options, the monitoring of which is essential to ensure remission and optimal response.

## Case presentation

A 19-year-old male presented to his optician in 2008 with complaints of progressive visual deterioration. At initial assessment, his best-corrected visual acuity (BCVA) was recorded as 6/6-1 in the right eye and 6/6+4 in the left eye. He was diagnosed with bilateral high myopia and subsequently referred to ophthalmology for further evaluation and to exclude the possibility of retinal detachment.

Ophthalmic examination revealed the anterior segments to be within normal limits bilaterally. Fundoscopic evaluation demonstrated myopic changes, including small optic discs and peripapillary atrophy in both eyes. The macula appeared normal bilaterally.

The patient was maintained under routine ophthalmic follow-up. However, two years later, he experienced a marked decline in visual acuity, with BCVA deteriorating to 6/36 in the right eye and counting fingers in the left eye. This significant reduction in visual function prompted further investigations, including magnetic resonance imaging (MRI) of the brain and anterior visual pathways, standard automated perimetry, and full-field electrodiagnostic testing.

Investigations

Standard visual field testing demonstrated variable visual field defects, with the most prominent loss occurring in the temporal fields of both eyes. Magnetic resonance imaging (MRI) of the brain revealed a large, avidly enhancing tumour mass centred on the clivus, measuring over 8 cm in maximum diameter. The lesion exhibited superior extension with effacement of the suprasellar cistern and invasion into the third ventricle. A substantial exophytic component was noted, extending laterally with mass effect on the right temporal lobe.

**Figure 1:**
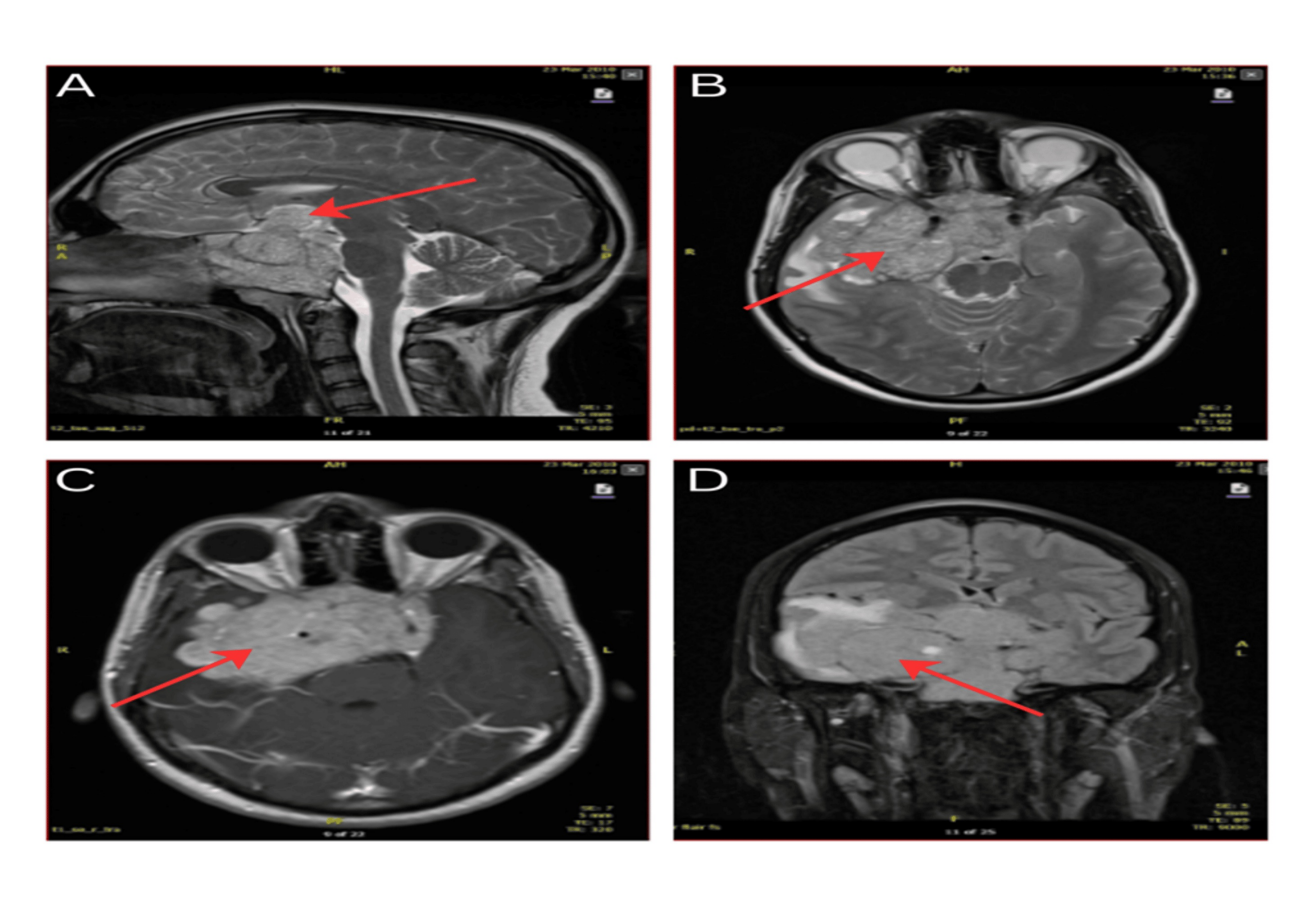
2010 MRI brain exhibiting pituitary mass (indicated by red arrows) with extension into right temporal lobe. A: Sagittal view; B and C: transverse view; and D: coronal view.

Posteriorly, the mass infiltrated the posterior aspect of the clivus and obliterated the pre-pontine cistern. This resulted in significant compression and displacement of adjacent brainstem structures, including the right side of the pons, midbrain, and right cerebral peduncle. Additionally, the tumour demonstrated complete encasement of the cavernous segments of both internal carotid arteries. On the right side, the tumour extended posteriorly to encase both the vertical and horizontal segments of the carotid artery, as illustrated in Figures 1A-1D.

Remarkably, the patient did not initially report any symptoms suggestive of raised intracranial pressure, such as headache, nausea, or vomiting, nor did he exhibit any focal neurological deficits on examination. There were no clinical signs indicative of pituitary hormone hypersecretion or deficiency. Systemic examination revealed no phenotypic features suggestive of growth hormone excess or deficiency (e.g. acromegaly), thyroid dysfunction (hypo- or hyperthyroidism), or adrenal insufficiency (Addisonian features) or excess (Cushingoid features). Furthermore, there were no symptoms of hypogonadism, including loss of libido, erectile dysfunction, or galactorrhoea.

A pituitary hormonal profile was obtained and demonstrated markedly elevated serum prolactin levels (794,560 mIU/L), consistent with a diagnosis of macroprolactinoma. Monomeric prolactin and macroprolactin levels measured through polyethylene glycol precipitation of serum were insignificant, ruling out interference by high molecular weight prolactin forms. Thyroid-stimulating hormone (TSH) was suppressed at 0.27 mIU/L, as detailed in Table 1. It is noteworthy that interpretation of the hormonal profile was limited by the patient’s inconsistent attendance at clinic appointments and irregular completion of blood tests, resulting in results obtained at varying time intervals.

**Table 1:** Hormonal profile of patient on initial presentation. IGF-1: insulin-like growth factor 1, FSH: follicle-stimulating hormone, LH: luteinizing hormone, TSH: thyroid-stimulating hormone, Free T4: free thyroxine.

Hormones	Results	Reference Range
Macroprolactin (mIU/L)	Not significant	
Monomeric prolactin (mIU/L)	794,560	45-375
IGF-1 (nmol/L)	41.9	15.1-46.5
FSH (IU/L)	0.3	1.6-18
LH (IU/L)	<0.1	2.0-18.0
TSH (mIU/L)	1.33	0.3-5.0
Free T4 (pmol/L)	12.5	11-23
Cortisol (nmol/L)	363 at (10:00)	
Male testosterone (nmol/L)	<0.4	8.4-28.7

Multiple interval scans were performed which showed unchanged appearance of the pituitary mass over the years. Serial monitoring of the prolactin levels revealed a down-trending but resistant pattern, as shown in Table 2.

**Table 2:** Trend of prolactin against cabergoline dosage in different time intervals.

Year	Monomeric Prolactin (mIU/L)	Cabergoline Dosage
2010	794,560	2 mg/day
2011	83,050	2 mg/day
2012	58,370	2 mg/day
2019	31,451	3 mg/day (self-titrated)
2024	15,372	2 mg/day

Management

Over the subsequent years, multiple multidisciplinary team (MDT) discussions were held at the regional pituitary centre. The consensus recommendation was to continue medical management with first-line dopamine agonist therapy (cabergoline), as the lesion demonstrated gradual radiological improvement and was deemed unsuitable for surgical intervention by neurosurgeons. The option of radiotherapy was presented several times to the patient; however, he felt he could not commit to the regular attendance of the regime of five days a week for five weeks.

Based on the patient's initial pituitary hormonal profile, hormonal replacement therapy was initiated and transdermal testosterone 2% gel (two actuations daily) for hypogonadism later changed to Nebido injections and then to Tostran. Six months after presentation, the patient developed central hypothyroidism (T4 10.8 pmol/L) and was also subsequently commenced on levothyroxine.

The initial endocrine team had started him on 14 mg of cabergoline per week (2 mg once daily) in 2010; however, the patient was unfortunately largely lost to follow-up due to poor compliance with clinic appointments. In 2019, he attended and mentioned he had self-medicated himself to 3 mg once daily (O.D) cabergoline, even going up to 10 mg O.D for a few days before experiencing hallucinations. He was heavily advised to titrate back down to 2 mg O.D, remaining on this regime for a total of 14 years with only four clinical reviews and three blood tests in the interim. Following ongoing specialist advice to continue 14 mg/week, the general practitioner in the community had renewed the off-label prescriptions during the years between clinical reviews.

In 2024, he re-presented to our own endocrinology service with a history of symptom progression, namely worsening headaches and fatigue, alongside reports of excessive spending and gambling since 2013. His dosage was still 14 mg/week of cabergoline at the time of review.

Outcome and follow-up

The most recent multidisciplinary team (MDT) meeting held in 2025 recommended a gradual reduction of the patient’s high-dose cabergoline therapy, given both the prolonged duration of treatment and the emergence of known dopamine agonist-related adverse effects. Of note, the patient had developed behavioural side effects (gambling, spending, etc.) consistent with dopaminergic dysregulation.

**Figure 2:**
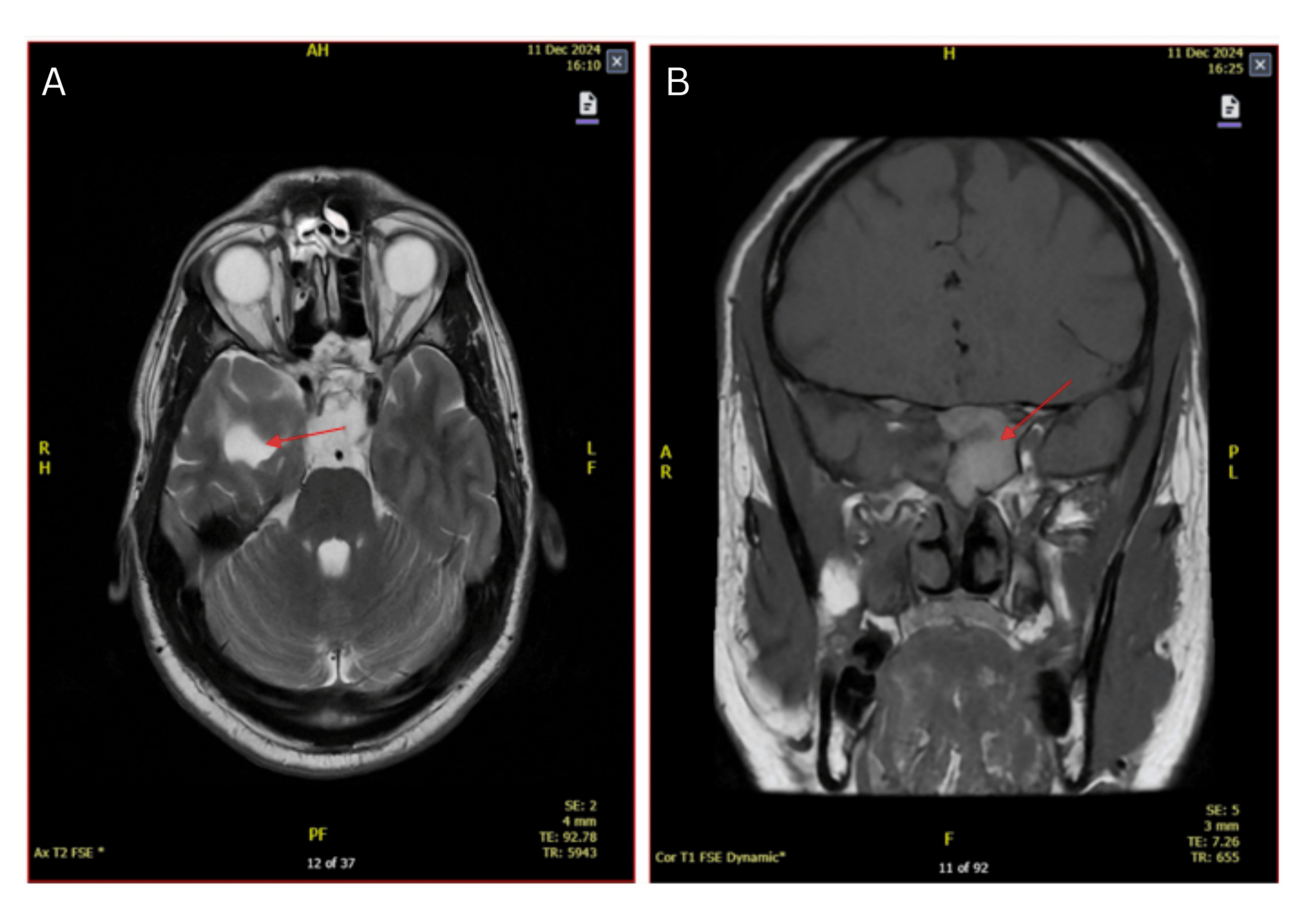
2024 MRI brain post-cabergoline treatment depicting partial reduction of pituitary tumour (shown with red arrows). A: Transverse view; and B: coronal view.

A follow-up MRI brain scan performed in December 2024 (Figures 2A, 2B) demonstrated a significant reduction of the tumour bulk, noting a residual nodular, solid, and contrast-enhancing component of the tumour involving the sphenoid sinus and bony skull base. In addition, gliotic changes were observed in the right inferior temporal lobe region. A portion of the tumour was also noted to extend into the left cavernous sinus.

A recent transthoracic echocardiogram, performed after over a decade of high-dose cabergoline therapy in the absence of routine cardiac monitoring, revealed no evidence of valvular heart disease or other structural abnormalities. Despite prolonged treatment with cabergoline at a cumulative dose reaching up to 14 mg weekly, the patient’s serum prolactin levels demonstrated only a partial biochemical response, decreasing from an initial level of 794,560 mIU/L to 15,000 mIU/L, a significant decrease but still above the normal range of 45-375 mIU/L. To investigate the possibility of a plurihormonal pituitary adenoma, a growth hormone suppression test was also conducted, which was negative, effectively ruling out co-secretion of growth hormone.

Cabergoline therapy is currently being down-titrated to 1 mg once daily, with a repeat MRI of the brain planned to monitor radiological progression. Notably, the patient has reported significant symptomatic improvement, particularly with resolution of compulsive behavioural disturbances previously attributed to dopaminergic overstimulation.

From a visual standpoint, recent ophthalmic assessment demonstrated best-corrected visual acuity of 6/5 in the right eye and 6/9 in the left eye with corrective lenses. Visual field testing revealed a right centrocecal relative scotoma and a dense left-sided temporal hemianopia, findings consistent with MRI evidence of tumoral involvement of the left optic chiasm and extension into the left cavernous sinus.

The patient is now under the care of the endocrinology team at his local district general hospital, with ongoing input from the regional tertiary care pituitary centre. Given the extensive invasive nature of the lesion, surgical resection is not feasible. The tumour has shown only partial responsiveness to maximal medical therapy with cabergoline. As such, consideration is being given to alternative treatment modalities, although this remains a significant therapeutic challenge in a young patient with a complex clinical course.

## Discussion

The presentation of this case is notable for the absence of high intracranial pressure symptoms. In this case, the patient only presented through his optician, who noticed an abrupt and significant decline in his visual fields and acuity. Typically, the excess prolactin from macroprolactinomas results in infertility, decreased libido, and neurological and visual changes ​[2]​. The patient’s macroadenoma measured over 8 cm in maximum diameter, extending into the third ventricle and imposing significant mass effect; however, the patient denied low libido, headaches, nausea, vomiting, or focal cranial nerve deficits. This should therefore prompt us to keep a low index of suspicion when encountering rapidly declining vision. On confirmation of a mass-inducing visual changes, improvement on neuroimaging and bloods should also be correlated with routine ophthalmologic assessments.

The patient was then discussed in an MDT, who agreed to a pharmacological approach. Cabergoline is generally the first-line pharmacological treatment of giant prolactinomas ​[7]​. In this case, our patient required unusually high doses of cabergoline, 14 mg/week, where the licensed maximum dose for pituitary adenomas is 4.5 mg/week, for over 10 years. Despite the high-dose therapy, only a partial reduction in tumour size and prolactin levels was achieved. The failure to bring prolactin into normal range or resolve the tumour radiologically is what qualifies this as a case of cabergoline resistance. Cabergoline has the highest efficacy; however, if a prolactinoma does not exhibit improvement in the first three to six months, it is unlikely to respond further ​[12]​ and may require consideration of alternative therapy. This highlights the importance of strict follow-up on commencement of cabergoline, as in this case, it may have led to earlier recognition of resistance. In cases of visual impairment and failure of pharmacology, surgery is often second line, and the most popular reason for surgical intervention remains to be resistance and intolerance to dopamine agonists ​[13]​. The possibility of surgery was discussed in multiple MDTs; however, each time neurosurgery deemed the tumour inoperable due to its location and invasion. Radiotherapy too may be considered as an adjunct to surgery; however, patient compliance to the regular regime must be assured which was not the case here ​[14]​. Should pharmacotherapy still be preferred despite DA resistance, alternative DA drugs or agents such as temozolomide, mammalian target of rapamycin (mTOR)/Akt inhibitors, and tyrosine kinase inhibitors may be explored ​[15]​. Alternative therapy for this patient was not truly explored due to the infrequent clinical reviews; however, with recent re-engagement, we are weaning down the cabergoline and look to consider quinagolide within the MDT instead.

The administration of cabergoline must weigh side effects against the risk of recurrence. Assuming adequate response, cessation should be considered after two years [12]. In this patient, regular outpatient appointments would have identified inadequate response earlier and, in light of the subsequent impulse control disorder, may have led to alternative therapies more promptly. Due to the patient's disengagement, any consideration of new medications was unfortunately very challenging. With long-term cabergoline use, the most common side effects to monitor for are postural hypotension, impulse control disorders (ICDs), and valvulopathy ​[2,16]​. The occurrence of valvulopathy is uncertain, with several studies pointing to no statistical association ​[17,18]​; however, it is still currently listed as a common side effect in the British National Formulary. It is worth noting that in this case, off-label high-dose cabergoline for 10 years yielded normal valvular structure on echocardiogram, something that may support the emerging research on the true cardiac impact of the drug. Our patient’s gambling and excessive spending is also in keeping with dopamine agonist-associated ICDs. These symptoms resolved on reduction of dosage, illustrating the need for psychiatric screening alongside cabergoline through suggested examples of the Patient Screening Questionnaire 9 and the Barratt Impulsiveness scale ​[19,20]​.

## Conclusions

The treatment of this patient’s macroprolactinoma remains suboptimal, and he requires serial multidisciplinary discussions. The upcoming plan is to repeat a pituitary MRI and blood profile to monitor response to 1 mg daily cabergoline. His prolactin levels remain above range, indicating sustained cabergoline resistance for which we may also consider alternative DA agents. We are also reconsidering radiotherapy within the MDT, although compliance poses an issue. We will then refer him back to the regional pituitary services for further advice.

Our patient's initial presentation of rapidly blurring vision hopefully underscores how diversely pituitary adenomas can present in clinical practice. For a condition which evolved to create so many consequences on the patient's life, it is therefore vital to keep in mind space-occupying lesions in ophthalmologic complaints. This case also highlights the key adverse effects when prescribing high-dose cabergoline, with particular focus on impulse control disorders, as they are strongly associated and difficult to monitor in the community. In the face of aggressive prolactinomas with incomplete biochemical control, we can appreciate the need to balance several management options (pharmacological, surgical, radiological, psychiatric) to create a tailored plan accounting for not just the presentation but also patient compliance.
